# Enhanced Recombinant Protein Production of Soluble, Highly Active and Immobilizable PNGase F

**DOI:** 10.1007/s12033-022-00464-6

**Published:** 2022-03-04

**Authors:** Noémi Kovács, Róbert Farsang, Márton Szigeti, Ferenc Vonderviszt, Hajnalka Jankovics

**Affiliations:** 1 Bio-Nanosystems Laboratory, Research Institute of Biomolecular and Chemical Engineering, University of Pannonia, 10 Egyetem Street, Veszprém, 8200 Hungary; 2 Translational Glycomics Research Group, Research Institute of Biomolecular and Chemical Engineering, University of Pannonia, 10 Egyetem Street, Veszprém, 8200 Hungary

**Keywords:** Capillary electrophoresis, *N-*glycan, SHuffle cells, PNGase F enzyme activity

## Abstract

High resolution analysis of *N*-glycans can be performed after their endoglycosidase mediated removal from proteins. *N-*glycosidase F peptide (PNGase F) is one the most frequently used enzyme for this purpose. Because of the significant demand for PNGase F both in basic and applied research, rapid and inexpensive methods are of great demand for its large-scale production, preferably in immobilizable form to solid supports or surfaces. In this paper, we report on the high-yield production of *N*-terminal 6His-PNGase F enzyme in a bacterial *Escherichia coli* SHuffle expression system. The activity profile of the generated enzyme was compared to commercially available PNGase F enzymes, featuring higher activity for the former. The method described here is thus suitable for the cost-effective production of PNGase F in an active, immobilizable form.

**Supplementary Information:**

The online version contains supplementary material available at 10.1007/s12033-022-00464-6.

## Introduction

PNGase F (Peptide-*N*_4_-(*N*-acetyl-beta-glucosaminyl) asparagine amidase from *Flavobacterium meningosepticum*) hydrolyzes a broad range of glycoproteins by readily partitioning the *N*-linked oligosaccharides from the protein. Therefore, it has been widely used in protein glycosylation studies. Due to its practical significance in high-throughput glycan analysis, there has been a great demand on the production of large amounts of pure and functional enzymes. However, PNGase F is secreted only in small amounts (0.1 mg/L culture, [1]) by its host. Recombinant expression in *Escherichia coli* may offer higher yield, but when expressed intracellularly, PNGase F is produced mainly in insoluble form [2]. To improve the yield of the soluble protein, PNGase F was expressed in the periplasm [3], but even this approach yielded only 8 mg/L culture of pure and active enzyme. Albeit higher levels of recombinant PNGase F were expressed in *Pichia pastoris* GS115 [4], the yeast partially glycosylated the enzyme during synthesis, producing a non-native form. Therefore, bacterial systems due to their controllable properties to achieve adequate protein expression levels may offer effective alternatives for rapid and inexpensive large-scale production of active PNGase F.

Extensive structural and functional studies of PNGase F [1, 5–7], including crystallographic analysis and site-directed mutagenesis of the 314 amino acid protein, identified three acidic residues (Asp-60, Glu-206, and Glu-118) to be essential for its activity [8]. These residues are in the cleft between the two domains of the protein and proper position of Asp-60 and Glu-206 are stabilized by disulfide bridges, suggesting that proper folding and S–S formation are crucial for the activity.

The *N*-terminal end of the polypeptide chain is located on the opposite side of the active site of the enzyme, so if PNGase F is attached to a solid support through that end, the catalytic center remains readily accessible [9, 10]. Oriented enzyme immobilization is one of the most promising processes to have a significant impact on deglycosylation efficiency. As it has been shown, GST tagged PNGase F anchored both on microcolumns or magnetic particles increased enzyme efficiency at least by a factor of two [9, 10].

In this paper, we present a workflow for high-yield bacterial production of the active form of PNGase F. The enzyme was expressed in an *E. coli* strain supporting the proper formation of disulfide bridges. For efficient purification and attachment to solid support, the PNGase F enzyme was His-tagged at its *N* terminus.

## Materials and Methods

### Chemicals and Reagents

The expression vector of pET17b was purchased from Novagen (Madison, Wisconsin, USA). Cloning *E. coli* strain TOP10 was from Invitrogen (Carlsbad, Canada). Purified BSA100X (10 mg/mL), restriction endonucleases AgeI HF and SacI HF, the expression host of SHuffle T7 Express Competent *E. coli* and one of the PNGase F enzymes were purchased from New England Biolabs (Ipswich, Massachusetts, USA). CarboClip PNGase F was ordered from Asparia Glycomics (San Sebastián, Gipuzkoa, Spain). T4 DNA ligase and PageRuler Prestained Protein Ladder were from Thermo Scientific (Waltham, Massachusetts, USA). Cells were cultured in LB Broth medium and LB Agar (Scharlau, Barcelona, Spain). Isopropyl β-d-1-thiogalactopyranoside (IPTG) was from BioChemica (Billingham, UK). EDTA-free Mini Complete Protease Inhibitor was purchased from Hoffman-La Roche (Basel, Switzerland). Template DNA (pGEX-3x-PNGase F) containing the coding sequence for PNGase F was kindly provided by Professor Miklos Guttman (University of Washington, Seattle, Washington, USA). DNA sequencing was performed by Macrogen Europe (Amsterdam, the Netherlands). The HiTrap Chelating Ni-affinity column was from GE Healthcare (Chicago, Illinois, USA). The human IgG (hIgG1) sample and all other chemicals were purchased from Sigma-Aldrich (St. Louis, Missouri, USA).

### Construction of the pET17b-6His-TEV-GSH-PNGaseF Plasmid

Template DNA (pGEX-3x-PNGase F) containing the coding sequence for the polypeptide sequence of the 41–354 segment of PNGase F (EC 3.5.1.52) was amplified by PCR using the following primers:

5′-ACATACCGGTGCTCCGGCAGATAATACCGTAAATATTAAAACATTCG-3′

(PNGaseF AgeI fw) and

5′-TACAGAGCTCTTAGTTTGTAACTACCGGAGCACTAATAGGTGTATTAC-3′ (PNGaseF SacI rev).

For protein expression the pET17b plasmid was modified by the substitution of the T7 tag with the coding sequence of an *N*-terminal 6-histidine tag, a TEV protease cleavage site, a glycine-serine-histidine (GSH) tripeptide and an AgeI restriction nuclease cleavage site, resulting in the *N*-terminal polypeptide HHHHHHENLYFQGSHTG fused to the inserted protein. The PCR product and a modified pET17b expression vector were digested by AgeI and SacI restriction enzymes and ligated by T4 ligase. Plasmid DNA was purified from TOP10 *E. coli* cells by Mini Plus Plasmid DNA Extraction System Kit (Viogene Biotek Corp., New Taipei City, Taiwan) and analyzed by DNA sequencing. DNA coding sequence of the 6His-PNGase F enzyme is presented in the Supplementary Material S1.

### Expression and Purification of 6His-PNGase F

SHuffle T7 Competent *E. coli* cells were transformed by the pET17b-6His-TEV-*pngaseF* plasmid using the standard protocol recommended by the manufacturer. 6His-PNGase F expression was performed by the inoculation of a 0.5 L LB medium supplemented with 100 µg/L ampicillin using 1% overnight starter culture. Cells were grown at 30 °C until OD_600_ reached 0.5–0.6, induced by 0.4 mM IPTG and incubated overnight at 16 °C with shaking. Cells from 0.5 L culture were suspended in 10 mL buffer ‘A’ (20 mM NaH_2_PO_4_, 500 mM NaCl, 25 mM imidazole, pH 7.5) supplemented with a protease inhibitor and incubated on ice for an hour. After the addition of 2% of glass beads (diameter 0.5 mm, Sigma-Aldrich), cells were disrupted by sonication. The remaining cell debris was partitioned by ultracentrifugation using 84,000×*g* on an Optima Max-XP Ultracentrifuge (Beckman Coulter, Brea, California, USA) for 30 min at 10 °C and the supernatant filtered through a 0.45 µm pore size syringe filter. A 5 mL HiTrap Chelating Ni-affinity column connected to an ÄKTA Start FPLC system (GE Healthcare) was pre-equilibrated with 10 mL buffer ‘A’ before loading the total filtrate (ca. 10 mL). The column was washed using a stepwise gradient of 20 mL buffer ‘A’ and 20 mL buffer ‘A’ containing 25 mM imidazole. The protein was eluted with 25 mL buffer ‘A’ containing 250 mM imidazole. The collected fractions were combined, diluted tenfold with buffer ‘A’ and repurified in three runs using the same purification method with the only exception of using 50 mM imidazole containing buffer in the washing step. The purity of the recombinant PNGase F was determined by SDS-PAGE, followed by concentration measurements at A_280_ using a molar extinction coefficient of 75,205/M/cm.

### Activity Measurement of 6His-PNGase F

The hIgG1 samples (10 µL of 2.0 mg/mL; 20 µg) were denatured prior to the endoglycosidase digestion at 80 °C for 10 min using 2.0 µL of a denaturation mixture (containing 0.6% sodium dodecyl sulfate (SDS), 12.5 mM dithiothreitol (DTT) and 0.06% NP40 detergent). 6His-PNGase F digestion was accomplished by adding the appropriate amount of the enzyme to the sample glycoprotein solution (in 20 mM ammonium acetate, pH 7.0) followed by incubation at 37 °C for 60 min. After the digestion step, 20 µL of labeling solution was added (containing 6.0 mM of 8-aminopyrene-1,3,6-trisulfonic acid (APTS), 100 mM of sodium cyanoborohydride in 1 M tetrahydrofuran (THF) and 24% of acetic acid) and labeled overnight at 37 °C, lid opened. Next, the excess dye was removed using 20 µL of tenfold concentrated M1 beads (Sciex, Framingham, Massachusetts, USA) and 185 µL of acetonitrile alternately in total of 4 wash cycles. Then, the APTS labeled sample was eluted using 100 µL HPLC grade water.

Capillary electrophoresis measurements were carried out on a PA 800 Plus instrument equipped with laser induced fluorescence (LIF) detector (Sciex) employing a 30 cm total length (20 cm effective length, 50 μm ID) bare-fused silica (BFS) capillary at 25 °C using 30 kV separation voltage in reversed polarity mode. The sample was introduced into the capillary by applying water for 1.0 psi for 5.0 s then the sample using 2.0 kV for 2.0 s. The results obtained were evaluated using the 32 Karat Software (version 10.1, Sciex).

## Results and Discussion

### Gene Design and Construction of the Plasmid Encoding 6His-PNGase F

The specific PNGase F gene for the deglycosylation experiments was designed to support oriented immobilization of the expressed protein via an oligo histidine tag. Considering the location of the active center of the endoglycosidase (PDB ID: 3PMS), the 6His-tag was fused to the *N* terminus of the enzyme. Beside the 6His-tag, an 11-amino-acids spacer was linked to the *N*-terminal end of the PNGase F. Even when the 6His-PNGase F enzyme is immobilized via the *N*-terminal 6His, it might be more accessible due to the spacer.

### Expression of 6His-PNGase F in SHuffle *E. coli*

The six cysteines in PNGase F should form three disulfide bridges when properly folded. Moreover, two of them play important role in stabilizing the active site [8]. To facilitate proper formation of the disulfide bridges, SHuffle T7 Express, an engineered *E. coli* strain that expresses disulfide-bond isomerase C (DsbC), was used as an expression host. The SHuffle T7 Express cells were transformed with a plasmid encoding the above-mentioned 6His-PNGase F and cultured in 0.5 L medium. The cells were harvested and subjected to purification steps including HiTrap Chelating Ni-affinity column chromatography. The yield of pure 6His-PNGase F was 46 mg per liter culture (Table 1 and Fig. S2). Compared with the previously reported yield of 8 mg/L [3], it is indicated that a reasonably high yield and purity can be obtained with our procedure.

**Table 1:** Total protein amount, yield and purity of the samples at each step of isolation of 6His-PNGase F

Purification step	Total protein [mg]^b^	Yield [%]	Purity [%]
Crude lysate^a^	66	100	53
Crude, filtered extract	53	80	57
Pooled peaks from affinity column	23^c^	35	97

^a^From 2.3 g of wet weight *E. coli* SHuffle cell pellet (obtained from 0.5 L of bacterial culture)

^b^Protein concentration determined from SDS-PAGE by densitometry using BSA (1.0 mg/mL) as a standard

^c^Determined by spectrophotometry from the absorbance measured at 280 nm

### Activity Measurement of 6His-PNGase F

The deglycosylation ability of 6His-PNGase F was tested on hIgG1 and its glycan profile was compared to those obtained under the same experimental conditions with commercially available enzymes (Fig. S3), suggesting that the produced PNGase F was properly folded and active, and cleaved *N*-glycans in a manner similar to other PNGase F enzymes.

**Fig. 1:**
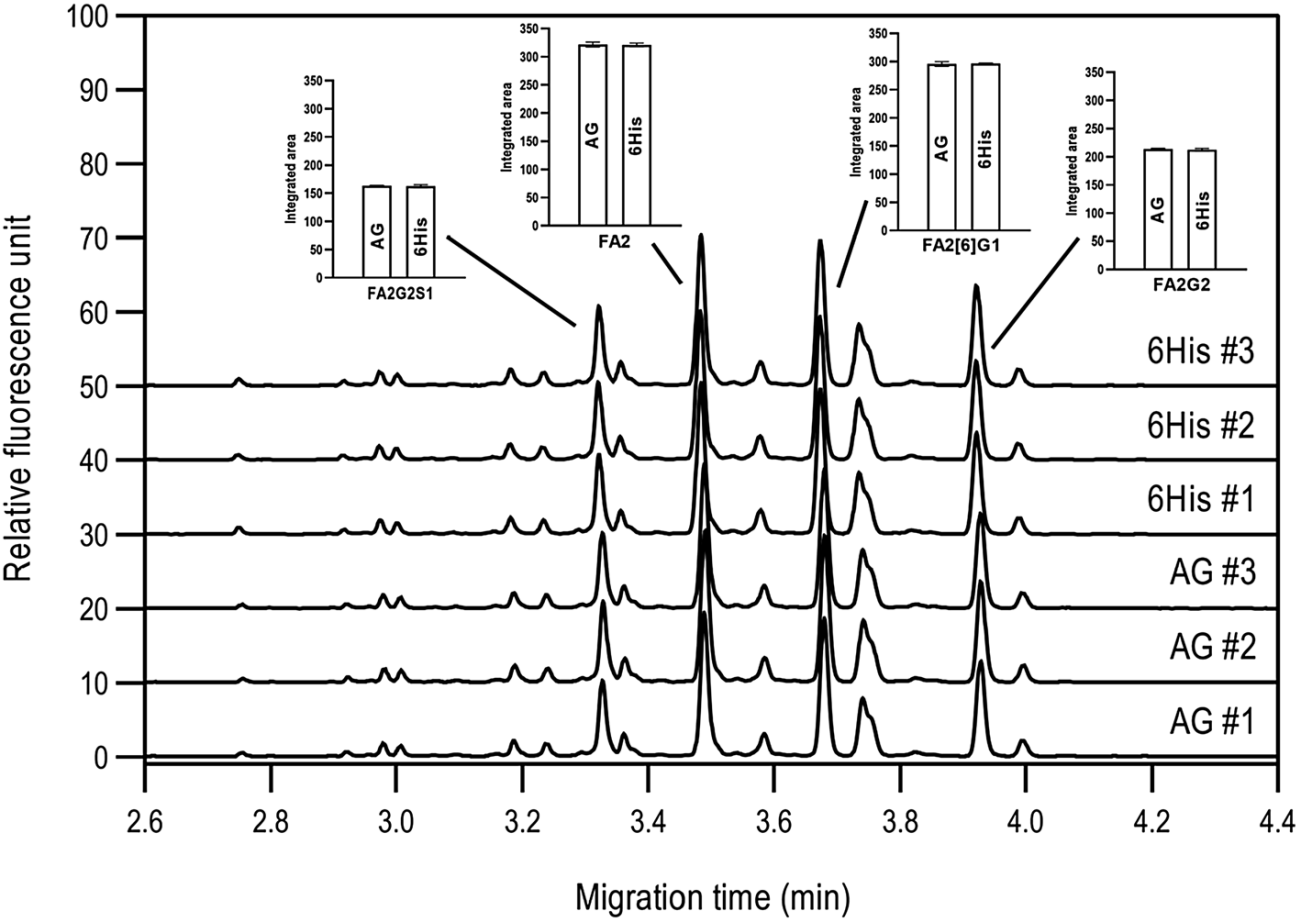
Comparison of *N-*glycan profiles of hIgG1 sample digested by 6His-PNGase F (denoted as 6His) and Asparia Glycomics 500 IUB activity PNGase F (AG) enzymes. The insets represent the integrated areas of four major peaks with the corresponding standard deviation from three parallel measurements. Separation conditions: 30 cm total length, 20 cm effective length BFS capillary. HR-NCHO separation gel buffer. Separation voltage: 30 kV in reverse polarity mode. Capillary temperature: 25 °C. Injection sequence: (1) 1.0 psi for 5.0 s HPLC grade water and (2) 2.0 kV/2.0 s sample

The activity of 6His-PNGase F was determined by comparing to a sample of a commercially available enzyme (CarboClip) of known activity and concentration. A stock solution of purified 6His-PNGase F at a concentration of 0.327 mg/mL containing 50% glycerol was used to prepare a dilution series using 20 mM Tris, 50 mM NaCl, 5.0 mM EDTA, at pH 8.0. One microliter of each PNGase F solution was added to a solution containing 20 µg denatured hIgG1 and incubated at 37 °C for 60 min. We found that the activity of 6His-PNGase F in the eightfold diluted sample matched well with the activity of 1.5 mU commercial PNGase F (Fig. 1). In the latter case, the lot analysis (Supplementary S4) suggests that 1.5 mU of enzyme corresponds to approximately 400 ng, whereas the 6His-PNGase F solution with nearly identical activity contains 41 ng enzyme. Since the difference between the molecular weights of the two enzymes is negligible (37.0 and 37.2 kDa for 6His-PNGase F and the commercial enzyme, respectively), it can be concluded that the activity of 6His-PNGase F is comparable to, and even higher than, that of the commercial enzyme under the conditions tested. Moreover, our preliminary results suggested that the 6His-PNGase F enzyme could be stored at − 20 °C in 50% glycerol at least for 24 months without decrease in activity. The recommended storage period for commercially available PNGase F enzymes varies between 1 and 2 years, suggesting that the storage period of 6His-labeled PNGase F enzyme without loss of activity meets or exceeds industry standards.

## Conclusion

In this work, an improved production efficiency of highly active PNGase F is shown to support large-scale *N-*glycan analysis. The HHHHHHENLYFQGSHTG peptide was fused to the PNGase F enzyme and the enzyme was produced in *E. coli* SHuffle cells to ensure proper formation of disulfide bridges and thus soluble and active protein. As a result of the optimization of protein expression and purification, a yield of 46 mg/L culture was achieved, which is higher than earlier published data in well-controlled and easily adaptable bacterial systems. The engineered PNGase F variant presented here, together with a protocol providing high protein yield and significant enzyme activity, ensures that the enzyme, which can be used in both soluble form and on solid supports, contributes to the growing demand for PNGase F.

## Supplementary Information

Below is the link to the electronic supplementary material.

Supplementary file1 (DOCX 193 kb)

## Abbreviations

hIgG1: Human immunoglobulin G1
PNGase F: Peptide-*N*_4_-(*N*-acetyl-beta-glucosaminyl) asparagine amidase
