# Supplementary material for: Enhanced Recombinant Protein Production of Soluble, Highly Active and Immobilizable PNGase F
Source: Mol Biotechnol. 2022 Mar 4;64(8):914–8. doi: 10.1007/s12033-022-00464-6 (PMC9259526; doi:10.1007/s12033-022-00464-6)
Supplement: Supplementary file 1 — Supplementary file1 (DOCX 193 kb) [file 12033_2022_464_MOESM1_ESM.docx]

**Supplementary Material**

**to**

**“Enhanced recombinant protein production of soluble, highly active and immobilizable PNGase F”**

**Supplementary S1**: DNA coding sequence of the 6His-PNGase F enzyme between the NdeI and SacI sites of pET17b expression plasmid:

atgcatcaccaccaccatcacgaaaacctgtattttcagggaagtcacaccggt*gctccggcagataataccgtaaatattaaaacattcgacaaagtaaaaaatgcctttggtgacggattgtcccaaagtgcagaaggaacctttacatttccggcagatgtaacaactgtaaaaacgattaagatgttcatcaaaaatgaatgtcctaataaaacctgtgatgaatgggatcgttatgccaatgtttatgtaaaaaataaaacaacaggagaatggtatgaaataggacgctttattactccatattgggtgggtacggaaaaattacctcgtggactggaaattgatgttaccgatttcaaatctttactgtccggaaatacagaacttaaaatttatacggagacttggttggccaaaggaagagaatacagtgtagactttgatattgtatatggtacaccggattataaatattcggcagtagtacctgtaatccaatataacaaatcatccattgatggtgttccttatggtaaagcacatacactgggattaaaaaagaatattcagttaccaacaaacacggaaaaagcttatcttagaactactatttccggatggggacatgccaagccatatgatgcgggaagcaggggctgtgcagaatggtgcttcagaacacatactatagcaataaataatgcgaatactttccaacaccagctgggtgctttaggatgttcagcaaaccctattaataatcagagtccgggaaattgggctcctgacagagcagggtggtgtccgggaatggcagtgccaacacgtatagatgtgttgaataactctttaacgggtagtacttttagttatgaatataagttccagagttggacaaacaacggaaccaatggagatgctttttatgcaatttccagttttgtgattgcaaaaagtaatacacctattagtgctccggtagttacaaac*taa


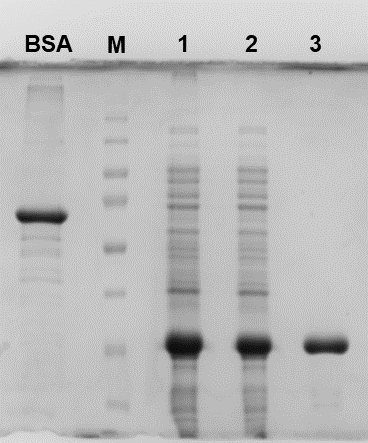


**Figure S2**: SDS-PAGE analysis of enzyme fractions obtained during purification. Lane BSA: 1.0 mg/mL BSA; lane M: molecular weight marker, the sizes of 8 bands detected are 170, 130, 100, 70, 55, 40, 35 and 25 kDa; lane 1: crude lysate; lane 2: crude filtered extract supernatant after cell disruption; lane 3: pooled peaks from the affinity column, purified enzyme.


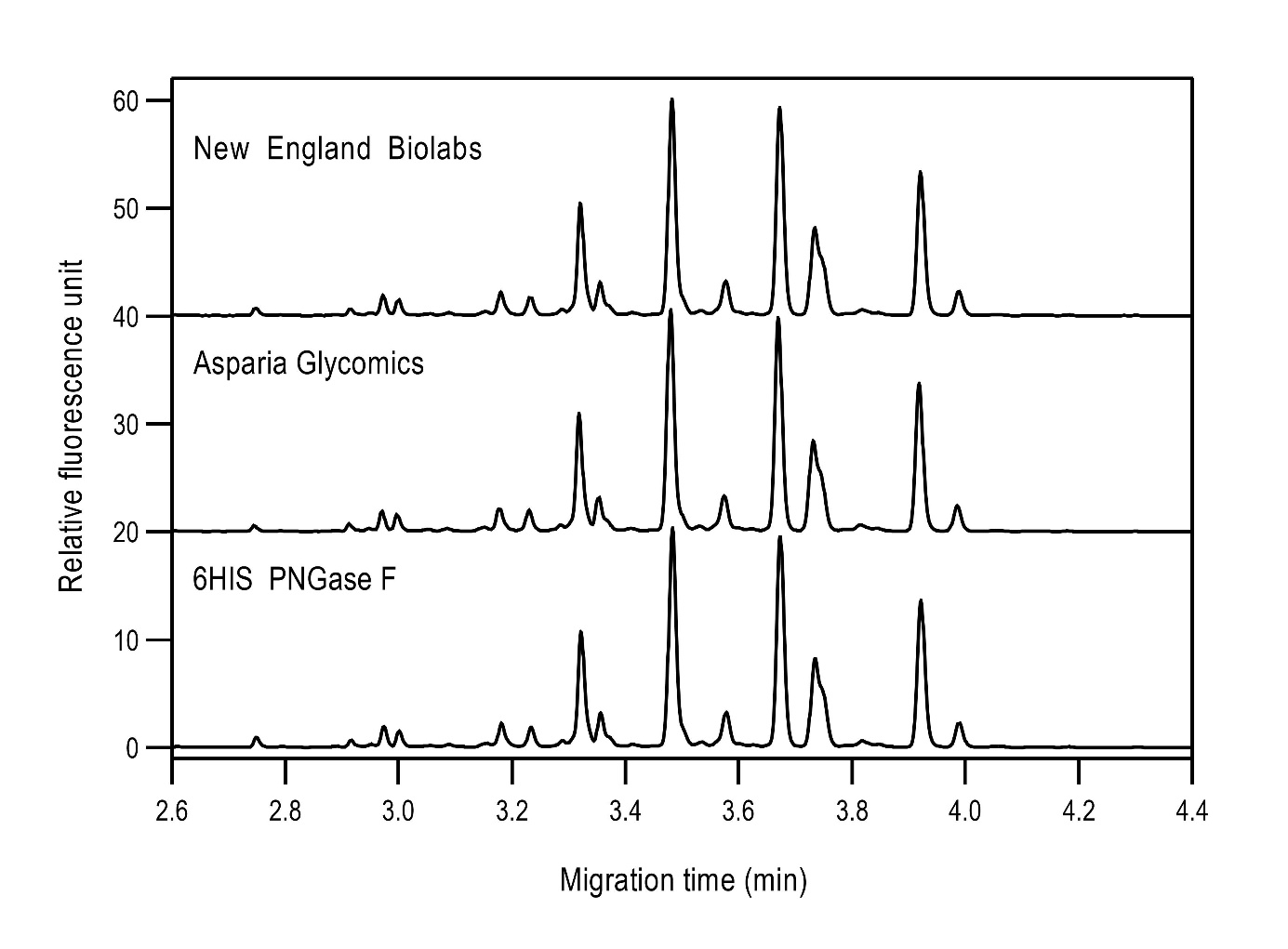


**Figure S3**: *N-*Glycan profiles of hIgG1 digested by 6His-PNGase F, Asparia Glycomics 500 IUB PNGase F, and New England Biolabs PNGase F enzymes. Separation conditions were the same as in Figure 1.

**Supplementary S4**: Calculation of the concentration of CarboClip PNGase F enzyme based on the description of the activity test (<https://aspariaglycomics.com/wp-content/uploads/2017/09/Analysis-CarboClip-lot-AD0CB0AG-500-mU.pdf>):

Vial containing estimated >500 mU and real 922 mU enzyme was supplemented with 150 µL water and a sample of this was further diluted 1:100. One µL of the diluted solution was added to a 10 µL glycoprotein sample and 2 µL of the 11 µL reaction mixture was sampled for SDS-PAGE from time to time. Based on the legend of the SDS-PAGE image, the lanes corresponding to the sampling contain 3 ng of PNGase F. Based on these, 1 mU real activity CarboClip enzyme corresponds to m = 3 ng / (922 mU / 150 µL / 100 / (2 µL / 11 µL)) = 268 ng enzyme. In our comparative deglycosylation experiment we used 1.5 mU CarboClip, which contains approximately 400 ng enzyme.
